# Sporadic foveolar-type adenoma in gastric body/antrum junction with gastritis cystica profunda

**DOI:** 10.1186/s12876-022-02285-y

**Published:** 2022-05-04

**Authors:** Jian Guan, Zhuo Li, Guihua Shen, Wenting Huang

**Affiliations:** 1 Pathology of Department, National Cancer Center/National Clinical Research Center for Cancer/Cancer Hospital, Chinese Academy of Medical Sciences and Peking Union Medical College, Beijing, 100021 China; 2 Pathology of Department, National Cancer Center/National Clinical Research Center for Cancer/Cancer Hospital and Shenzhen Hospital, Chinese Academy of Medical Sciences and Peking Union Medical College, Shenzhen, 518116 China

**Keywords:** Stomach, Foveolar-type adenoma, Stomach neoplasms, Metaplasia

## Abstract

**Background:**

Sporadic gastric foveolar-type adenomas are extremely rare and are usually small, flat or slightly raised lesions that occur in the oxyntic mucosa.

**Case presentation:**

We reported here a case of a 70-year-old female with a sporadic gastric foveolar-type adenoma occurring in the mucosa at the junction of the gastric body/antrum. The adenoma was a protruding lesion of 2 × 1.8 cm sized, causing symptoms of upper gastrointestinal bleeding, and the basal and surrounding mucosa showed pseudopyloric gland metaplasia without atrophy, intestinal metaplasia, H. pylori infection, or active inflammation. It had somatic mutations in both APC and KRAS genes.

**Conclusions:**

This is the first reported case of a large sporadic gastric foveolar-type adenoma that occurred in the mucosa of pseudopyloric gland metaplasia and with Gastritis Cystica Profunda, which modify our understanding of the morphological features and molecular underpinnings of this type of lesion.

## Background

Sporadic gastric foveolar-type adenoma is an extremely rare gastric lesion, usually a small, flat polypoid lesion that occurs in the oxyntic mucosa [1]. Compared with other gastric adenomas, it occurs in the relatively healthy gastric mucosa with a lower risk of progression to cancer. Its pathogenesis is considered to be different from the “inflammation-atrophy-precancerous pathway” [2]. Most of the gastric foveolar-type adenomas were related to the hereditary tumor syndrome, such as familial adenomatous polyposis (FAP) and gastric adenocarcinoma and proximal gastric polyposis (GAPPS) [3]. The sporadic cases reported in the literature are very rare, and the molecular mechanism of their formation is not clear. The article reported here a case of a large sporadic gastric foveolar-type adenoma. Unlike previous reports, it occurred in the gastric body-antrum mucosa of pseudopyloric gland metaplasia and combined with gastritis cystica profunda.

## Case presentation

**Fig. 1:**
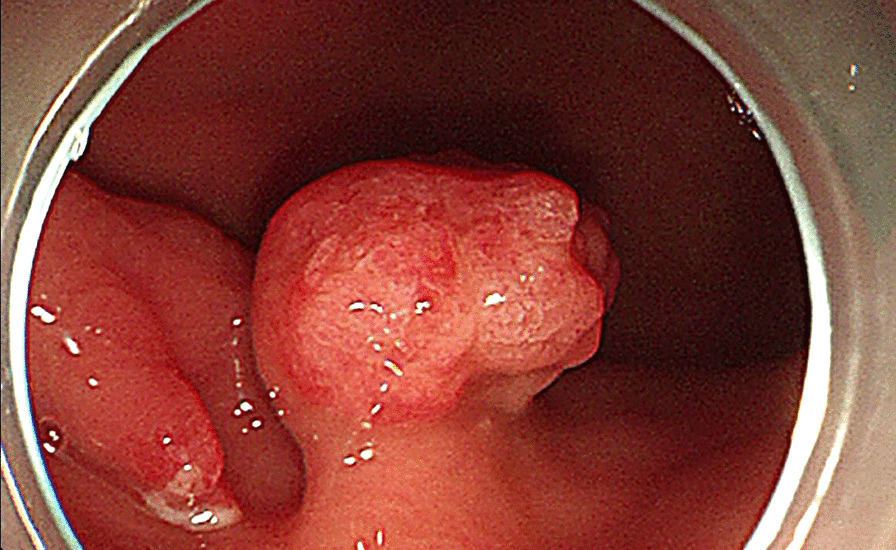
Gastroscopy findings: There were two protruding lesions in the mucosa at the junction of the gastric body/antrum (50 cm from the incisors). The larger one (right side) was a broad-based polyp with a papillary or gyrus-like appearance in the surface; the smaller one (left side) was a flat polyp

A 70-year-old female patient was referred to our hospital for "hematemesis for 10 days". The volume of hematemesis was about 150 ml. The patient had melena, but no fresh blood in the stool. Gastroscopy showed there were two wide-base polyps in the greater curvature of gastric body/antrum junction (about 50 cm from the incisor teeth). The larger one was 2 × 1.8 cm in size, with a papillary or gyrus-like appearance in the surface; the smaller one was 1 × 1 cm in size and was slightly flat (Fig. 1). And there was another wide base polyp sized 0.8 × 0.8 cm in the minor curvature of the cardia (about 41 cm from the incisor). The gastric body-antrum mucosa showed no atrophic gastritis appearance.

The patient had no history of gastrointestinal polyps and Helicobacter pylori (H. pylori) eradication therapy. She had no family history of tumors and six offspring were in good health.

The patient underwent endoscopic mucosal dissection (ESD) on the larger polyp at the junction of the gastric body/antrum and endoscopic mucosal resection (EMR) on the other smaller polyps. The operation process went smoothly, and the patient's postoperative condition was stable.

### Gross examination

The ESD specimen of the larger polyp at the junction of the gastric body/antrum showed a broad-based protruding lesion with a size of 2.3 × 1.3 × 1.3 cm. There were no ulcers and necrosis on the surface. The cut surface was solid, gray-pink, and soft in texture. The lesion was confined to the mucosal layer and the nearest distance to the mucosal lateral margin was 0.5 cm. The smaller polyp at the junction of the gastric body/antrum and the polyp in the subcardia area were both sessile polyps, the size of which was 0.8 × 0.5 × 0.3 cm and 0.5 × 0.4 × 0.2 cm, respectively.

### Microscopic examination

**Fig. 2:**
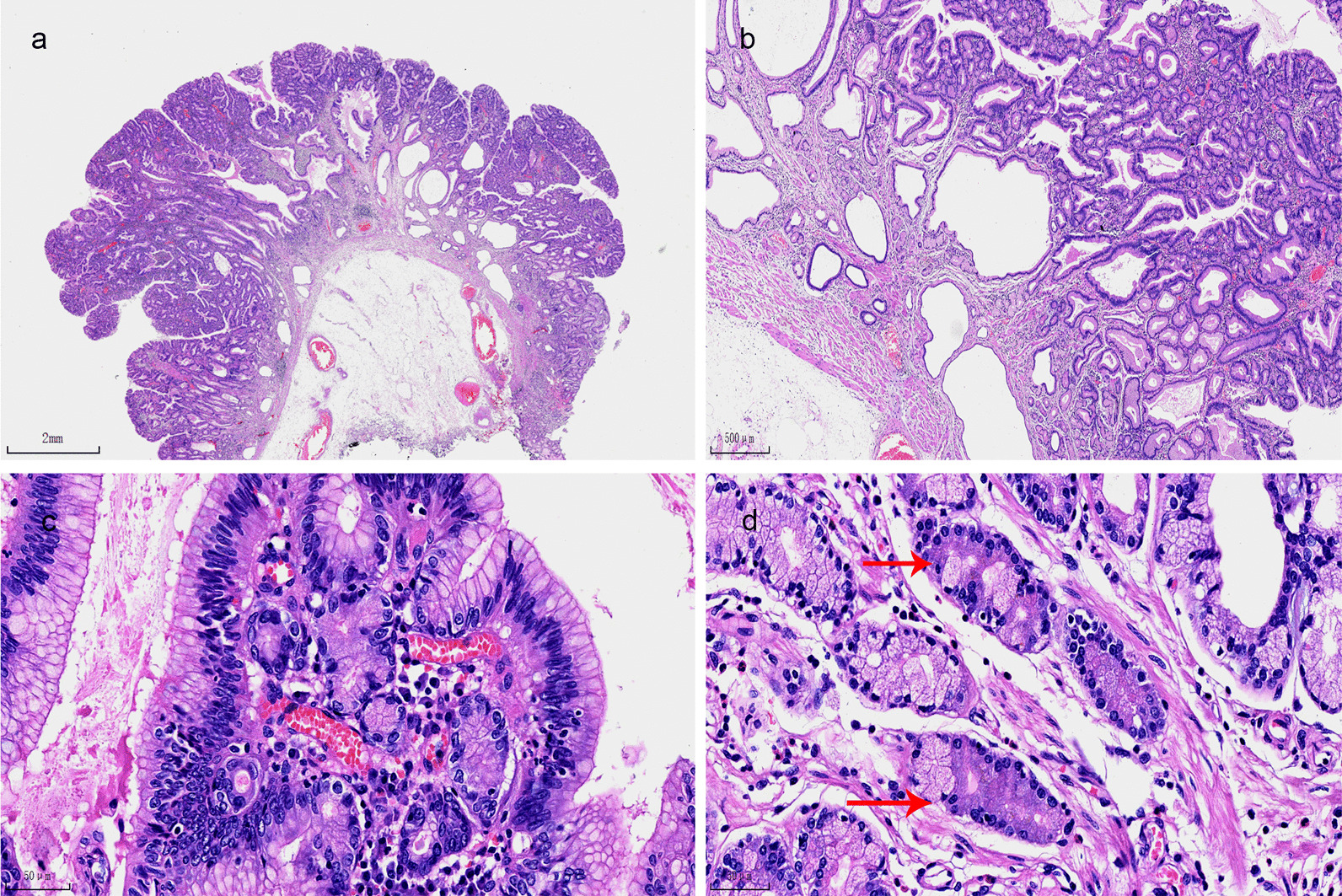
Photomicrographs of the larger polyp at the junction of the gastric body/antrum, H&E staining: a protruding mucosal lesion showed a tubulovillous-like structure, ×15 (**a**); the surface glands proliferated densely, and the glands close to the muscularis mucosa expanded and locally pushed toward the muscularis mucosa, ×40 (**b**); the tumor cells were columnar epithelium with apical mucin caps in the cytoplasm, the nucleus was enlarged and elongated, located at the base of the cytoplasm, ×400 (**c**); the mucosa of the tumor base and surrounding were pyloric gland-like morphology, and a small amount of basophilic main cells were scattered in the glands (shown by arrow), ×400 (**d**)

The larger polyp at the junction of the gastric body/antrum was a 0-Is protruding type lesion (Fig. 2a). The tumor consisted of dysplastic columnar epithelia arranged in a tubular-villous pattern. The surface glands were densely proliferated while the basal glands were expanded (Fig. 2b). The tumor cells were mainly columnar, and the nuclei were of the same size and located at the basal part of the gland with rarely stratified structure. Folded papillae could be seen in some expended glands. Compared with the surrounding normal gastric foveolar epithelium, the tumor cells were enlarged and the nuclei were elongated and densely arranged. There were apical mucin caps in the cytoplasm (Fig. 2c). High-grade areas and an invasive component were not seen. There were infiltrating inflammatory cells but no edema and proliferation of smooth muscle bundles in the stroma. The basal dilated glands were covered with low columnar, cubic, or flat epithelium without any dysplasia, locally pushing to the muscularis mucosa without stromal reaction. The surrounding mucosa showed the morphology of pyloric glands, with a few main cells scattered in a small number of glands, lack of parietal cells (Fig. 2d). There was no atrophy, intestinal metaplasia, H. pylori infection, or active inflammation in the mucosa.

**Fig. 3:**
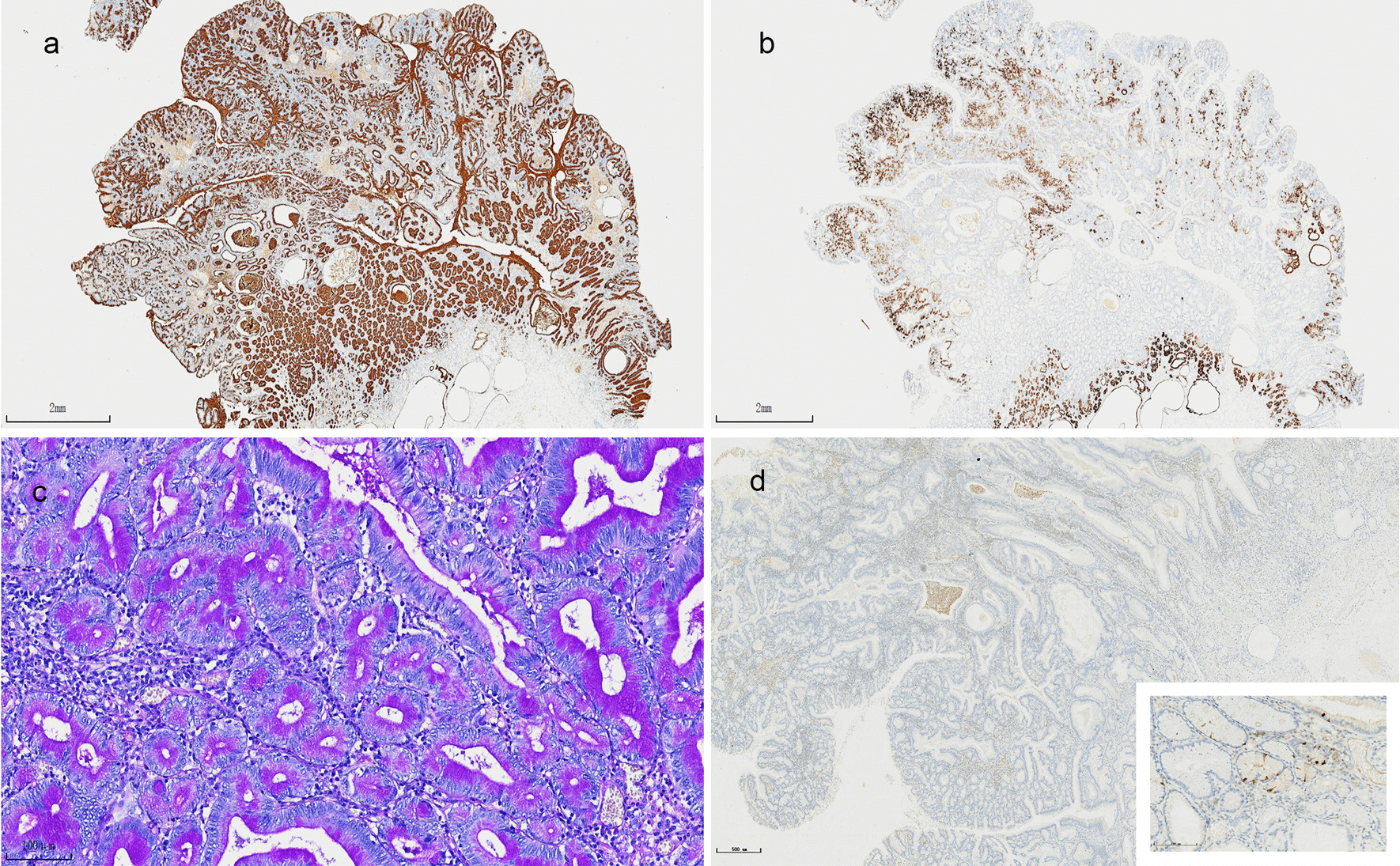
Photomicrographs of the larger polyp at the junction of the gastric body/antrum, immunohistochemical and special staining. Immunohistochemical staining showed diffuse staining of MUC5AC, ×15 (**a**), MUC6 was partially stained in the adenoma part, but diffuse staining in the tumor base and surrounding mucosa, ×15 (**b**); PAS staining showed intraplasmic mucus, ×200 (**c**); Gastrin has no positive expression in the tumor and basal mucosa, ×40 (**d**), a small amount of gastrin expressing cells could be seen in the surrounding mucosa, ×200 (**d** inset)

The immunohistochemistry staining of MUC5AC showed diffuse cytoplasmic staining (Fig. 3a), while MUC6 showed focal cytoplasmic staining (Fig. 3b). There was a small amount of CDX2 staining in the superficial area of the tumor. No staining of MUC2 was observed. PAS (Periodic acid-Schiff) staining of the larger polyp showed that there were purple apical mucin caps in the cytoplasm, although being reduced compared with the surrounding normal gastric foveolar mucosa (Fig. 3c). The Ki-67 index was higher on the surface of the tumor (10% +), but lower in the expanded glands at the base. Gastrin staining showed no positive expression in the tumor and tumor basal mucosa, but a few gastrin-positive cells were seen in the mucosa around the tumor (Fig. 3d).

A diagnosis of gastric foveolar-type adenoma with low-grade dysplasia was rendered; the base of the tumor was accompanied by gastritis cystica profunda (GCP).

The other two polyps at the junction of the gastric body/antrum and the subcardia area were similar in morphology. They were composed of foveolar epithelium without dysplasia, arranged with enlarged and twisted glands accompanied by stroma edema and inflammatory cell infiltration. The morphology of these two lesions was consistent with gastric hyperplastic polyps.

### Genetic analysis

A somatic genetic test was performed on the larger polyp at the junction of the gastric body/antrum. We analyzed 20 genes related to gastric tumors and polyps through next-generation gene sequencing (NGS) and found APC and KRAS gene mutations in the adenoma tissue. See “Appendix” for the list of genes tested and specific mutation forms.

## Discussion

Most of the gastric foveolar-type adenomas are related to hereditary tumor syndromes, such as familial adenomatous polyposis (FAP) and gastric adenocarcinoma and proximal gastric polyposis (GAPPS), and only a few sporadic cases have been reported [4, 5]. FAP is a series of syndromes including gastrointestinal polyps, cancer, and extracolonic lesion caused by germline mutations of the APC gene [3, 6]. GAPPS is considered to be a subtype of FAP, but has unique clinical manifestations [7]. The patient only underwent somatic mutation testing of the tumor, without performing germline mutation testing at the same time for personal reasons. Considering her age at onset (70 years old) and the absence of other gastrointestinal and systemic related lesions other than the gastric lesions, and no family history, we speculated that she was a patient of a sporadic gastric foverolar-type adenomas having concurrent presence of APC and KRAS somatic gene mutations. In addition, the somatic mutation abundance of APC and KRAS gene was 23.49% and 14.38%, respectively, which also tended to suggest that it was a sporadic case. Due to the rare occurrence, research on the molecular basis of sporadic gastric foveolar-type adenomas is limited. Some study about FAP-related gastric foveolar-type adenomas has reported somatic mutations in APC and KRAS [8]. It indicates that sporadic and syndromic gastric foveolar-type adenomas might share common genetic aberrations. We also tested several other gastric tumor and polyp related genes, involving PI3K/Akt, MAPK and Wnt/beta-catenin pathways (listed in the “Appendix”), but no other related gene mutations were found.

Some researchers believe that most adenomas are mucosal polypoid dysplasia that develops from chronic gastritis [9]. Except for being related to hereditary tumor syndrome, sporadic adenomas occurring in a non-inflammatory background are rare. Unlike the intestinal-type adenomas and pyloric gland adenomas related to H. pylori infection and atrophic gastritis [10], gastric foveolar-type adenoma often develops in healthy gastric mucosa and mostly occurs in the oxyntic gastric compartment (body/fundus), no matter it is sporadic or systemic. The case reported here was an adenoma that occurred in the mucosa at the junction of the gastric body/antrum, without obvious gland atrophy and H. pylori infection in the background. The adenoma tissue was diffusely positive for MUC5AC, while MUC6 was focally positive, indicating that the epithelium that composed the adenoma was a gastric foveolar phenotype. While the glands at the base of the adenoma showed diffuse staining of MUC6 but no gastrin staining, composed of columnar epithelium with only a small number of main cells scattered, indicating that it was a pseudopyloric gland metaplasia. Pseudopyloric gland metaplasia is considered to be related to inflammation and atrophy-related gastric mucosal repair [11], but in this case, there was no clear gastritis under endoscopy and microscope. The possible reason was that the patient was older (70 years old), due to the pyloric glands metaplasia of oxyntic mucosa with age, the line of gastric corpus/antrum junction was pushing forward the fundus of stomach. The morphology of this case enriches the performance of gastric foveolar-type adenoma, which can occur in the gastric mucosa of pseudopyloric gland metaplasia.

Sporadic gastric foveolar adenomas are extremely rare. The cases reported before are usually small, flat, or even depressed lesions located in the mucosa of gastric oxyntic glands. We reported here a protruding lesion with a maximum diameter of 2.3 cm, which caused symptoms of gastrointestinal bleeding. Recently, there is a study about a type of sporadic gastric foveolar-type adenoma with a raspberry-like appearance [5], which is considered as a special subtype of sporadic foveolar-type adenoma. The cases they reported were all located in the upper or middle stomach, and the mean lesion size was quite small at 3.2 ± 2.6 mm. Our case is the first case of a large-sized gastric foveolar adenoma developing at the junction of the gastric body/antrum.

The case also showed gastritis cystica profunda at the base of the tumor. The occurrence of gastritis cystica profunda is considered to be related to iatrogenic (surgical operation) infection, chronic ischemia, or inflammation, showing prolapse of the mucosa and glands to the muscularis mucosa and even the submucosa [12]. The deep-seated cystic gastritis at the base of the adenoma reported here may be related to the excessive size of the tumor causing chronic ischemia at the base. Gastritis cystica profunda at the base of adenomas is easily confused with adenocarcinoma [4]. But the deeply located glands were lack of cytological atypia and stromal desmoplasia, which could be differentiated from adenocarcinoma.

In summary, we reported a valuable case of sporadic gastric foveolar-type adenoma which occurred at the junction of gastric corpus/antrum in an elderly patient. The tumor was large in size and caused symptoms of upper gastrointestinal hemorrhage and accompanied by gastritis cystica profunda. This is different from the previously reported cases, which expands the clinical manifestations of gastric foveolar-type adenoma and increases the understanding of its morphology and molecular basis.

## Data Availability

All data generated or analyzed during this study are included in this article.

## Appendix

The list of gene analyzed by NGS:

**Table:**

APC	ATM	BRAF	CTNNB1	EGFR
ERBB2	ERBB4	FGFR2	FGFR3	KRAS
MAP2K1	MET	NF1	NRAS	NTRK1
NTRK3	PIK3CA	PTEN	SMAD4	TP53

Somatic mutation test results:

**Table:**

Gene ID	Analysis result	Location	Transcript	Mutation abundance (%)	Clinical significance
KRAS	p.Q61H (c.183A>C)	EX3	NM_033360.2	14.38	Pathogenic/Likely pathogenic
APC	p.S1465Wfs*3 (c.4393_4394del)	EX16	NM_000038.5	23.49	Pathogenic/Likely pathogenic

## Abbreviations

FAP: Familial adenomatous polyposis
GAPPS: Gastric adenocarcinoma and proximal gastric polyposis
ESD: Endoscopic mucosal dissection
EMR: Endoscopic mucosal resection
PAS: Periodic acid-Schiff
GCP: Gastritis cystica profunda
NGS: Next generation sequencing
